# Commercial Formulates of *Trichoderma* Induce Systemic Plant Resistance to *Meloidogyne incognita* in Tomato and the Effect Is Additive to That of the *Mi-1.2* Resistance Gene

**DOI:** 10.3389/fmicb.2019.03042

**Published:** 2020-01-31

**Authors:** Miriam Pocurull, Aïda M. Fullana, Miquel Ferro, Pau Valero, Nuria Escudero, Ester Saus, Toni Gabaldón, F. Javier Sorribas

**Affiliations:** ^1^Department of Agri-Food Engineering and Biotechnology, Universitat Politècnica de Catalunya, Barcelona, Spain; ^2^Bioinformatics and Genomics Program, Centre for Genomic Regulation, Barcelona Institute of Science and Technology, Barcelona, Spain; ^3^Department of Experimental and Health Sciences, Universitat Pompeu Fabra, Barcelona, Spain; ^4^Catalan Institute for Research and Advance Studies (ICREA), Barcelona, Spain

**Keywords:** *Cucumis sativus*, induced resistance, nematode virulence, *Pochonia chlamydosporia*, root-knot nematodes, *Solanum lycopersicum*

## Abstract

*Meloidogyne* is the most damaging plant parasitic nematode genus affecting vegetable crops worldwide. The induction of plant defense mechanisms against *Meloidogyne* in tomato by some *Trichoderma* spp. strains has been proven in pot experiments, but there is no information for tomato bearing the *Mi-1.2* resistance gene or for other important fruiting vegetable crops. Moreover, *Trichoderma* is mostly applied for managing fungal plant pathogens, but there is little information on its effect on nematode-antagonistic fungi naturally occurring in soils. Thus, several experiments were conducted to determine (i) the ability of two commercial formulates of *Trichoderma asperellum* (T34) and *Trichoderma harzianum* (T22) to induce systemic resistance in tomato and cucumber against an avirulent *Meloidogyne incognita* population in split-root experiments; (ii) the effect of combining T34 with tomato carrying the *Mi-1.2* resistance gene to an avirulent *M. incognita* population in sterilized soil; and (iii) the effect of combining T34 with tomato carrying the *Mi-1.2* resistance gene to a virulent *M. incognita* population in two suppressive soils in which *Pochonia chlamydosporia* is naturally present, and the effect of T34 on the level of *P. chlamydosporia* egg parasitism. Both *Trichoderma* formulates induced resistance to *M. incognita* in tomato but not in cucumber. In tomato, the number of egg masses and eggs per plant were reduced by 71 and 54% by T34, respectively. T22 reduced 48% of the number of eggs per plant but not the number of egg masses. T34 reduced the number of eggs per plant of the virulent *M. incognita* population in both resistant and susceptible tomato cultivars irrespective of the suppressive soil, and its effect was additive with the *Mi-1.2* resistance gene. The percentage of fungal egg parasitism by *P. chlamydosporia* was not affected by the isolate T34 of *T. asperellum*.

## Introduction

The root-knot nematodes (RKN), *Meloidogyne* spp., are the most damaging obligate plant-endoparasitic nematode worldwide in a wide range of plant species (Jones et al., 2013). Among the more than 100 species included in this genus, the tropical RKN species, *Meloidogyne arenaria*, *Meloidogyne incognita*, and *Meloidogyne javanica*, cause the majority of vegetable yield losses (Hallman and Meressa, 2018). For instance, maximum yield losses reported for fruiting solanaceous and cucurbit crops, the most cultivated worldwide, range from 30 to 100% (Giné et al., 2014, 2017; López-Gómez et al., 2014; Seid et al., 2015; Giné and Sorribas, 2017; Hallman and Meressa, 2018). Despite that several methods for control are available (Nyczepir and Thomas, 2009), most producers rely on the use of chemical nematicides (Djian-Caporalino, 2012; Talavera et al., 2012). Nonetheless, due to the risks and impacts on human health and the environment, its use must be reduced in favor of alternative methods according to legislative regulations, such as the European Directive 2009/128/EC. Sustainable and safe alternatives are required, such as plant resistance and biological control (Hallmann et al., 2009; Williamson and Roberts, 2009).

Plant resistance is defined as the ability of a plant to suppress infection, development, and/or reproduction of plant parasitic nematodes (Roberts, 2002). Resistance can be conferred by one or a few specific genes or quantitative trait loci (Williamson and Roberts, 2009) or be induced by microorganisms (Hallmann et al., 2009; Schouten, 2016). Plant resistance conferred by resistance genes (*R*-genes) is an effective and economically profitable control method against the tropical RKN species (Sorribas et al., 2005). However, the availability of commercial fruiting vegetable-resistant cultivars and rootstocks is currently restricted to solanaceous crops such as tomato, pepper, and aubergine. Therefore, the continuous cultivation of plant germplasm carrying the same *R*-gene will lead to the selection of virulent nematode populations (Verdejo-Lucas et al., 2009; Thies, 2011; Ros-Ibáñez et al., 2014; Expósito et al., 2019). Some fungal and bacterial species are able to induce resistance against RKN in vegetable crops (Hallmann et al., 2009), including some strains of *Trichoderma* spp., i.e., *Trichoderma asperellum* strain 203, *Trichoderma atroviride* strain T11, and *Trichoderma harzianum* strain T-78 in tomato (Sharon et al., 2009; de Medeiros et al., 2017; Martínez-Medina et al., 2017). Several *Trichoderma* spp. strains are approved by the EU legislation for controlling plant diseases caused by fungi but none of them for those caused by plant-parasitic nematodes.

The possibility of using *Trichoderma* spp. to induce resistance to RKN has been studied in susceptible tomato cultivars (Sharon et al., 2009; de Medeiros et al., 2017; Martínez-Medina et al., 2017) but never on tomato carrying the *Mi-1.2* resistance gene. Induction of resistance in plants carrying *R-*genes could contribute to limit the selection of virulent nematode populations and thus enhancing the resistance durability. On the other hand, if resistance can be induced in plant species for which no commercial RKN-resistant cultivars or rootstocks are available, such as cucurbits, or against virulent nematode populations, primed plants could be included in rotation schemes to manage RKN and reduce crop yield losses.

*Trichoderma* is a cosmopolitan genus of filamentous fungi in the order Hypocreales, with a flexible lifestyle that includes endophytic, saprophytic, and facultative mycoparasitic capabilities. Thus, *Trichoderma* spp. might limit growth of other soil microorganisms by predation or resource competition, including nematode antagonistic fungi such as *Pochonia (Metacordyceps) chlamydosporia*. This fungal species is frequently isolated from RKN eggs produced in vegetable crop roots cultivated in northeastern Spain (Giné et al., 2012) and has been reported as the main biotic factor responsible for soil suppressiveness to RKN in this area (Giné et al., 2016). In addition, it has been reported that some *P. chlamydosporia* strains can induce systemic resistance in tomato plants (Ghahremani et al., 2019). Consequently, the proper use of *Trichoderma* must consider the possible side effect on fungal nematode antagonists present in soils. Previous studies have shown that the effects of *Trichoderma* to *P. chlamydosporia* can vary depending on the analyzed fungal strains. For example, some harmful effects such as a reduction in mycelial growth due to volatile compounds produced by a strain of *Trichoderma* spp. from Brazil (Ferreira et al., 2008) or mycelium lysis by a strain of *T. harzinaum* from the Netherlands (Kok et al., 2001) have been reported. On the other hand, non-observable effects on percentage of RKN-parasitized eggs by *P. chlamydosporia* due to a strain of *T. harzianum* from Cuba was noticed (Puertas et al., 2006). However, as far as we know, there is no information regarding the effect of commercial formulates of *Trichoderma* spp. on the level of fungal egg parasitism by natural occurring antagonists in soil to avoid side effects.

Thus, in this work, several experiments were conducted to determine (i) the ability of commercial formulates of strains T34 of *T. asperellum* [T34 Biocontrol (10^12^ cfu kg^–1^); Biocontrol Technologies S.L.] and T22 of *T. harzianum* [Trianum P (10^9^ cfu g^–1^); Koppert] to induce systemic resistance in tomato and cucumber against *M. incognita* in split-root experiments; (ii) the effect of combining T34 with tomato carrying the *Mi-1.2* resistance gene to an avirulent *M. incognita* population in sterilized soil; and (iii) the effect of combining T34 with tomato carrying the *Mi-1.2* resistance gene to a virulent *M. incognita* population in two suppressive soils where *P. chlamydosporia* is naturally present, as well as the effect of T34 on the level of *P. chlamydosporia* egg parasitism.

## Materials and Methods

### Plants, Fungi, and Nematodes

Susceptible (*mi/mi*) tomato cv. Durinta, resistant (*Mi/mi*) tomato cv. Monika (Cortada et al., 2008), and cucumber cv. Dasher II were used for this study. For the split-root and the combination of plant resistance with T34 to an avirulent *M. incognita* population experiments, seeds were surface sterilized following the procedure described in Ghahremani et al. (2019).

The commercial formulates of *T. asperellum* T34 (T34 Biocontrol; Biocontrol Technologies S.L.) and of *T. harzianum* T22 (Trianum P; Koppert) were used. These *Trichoderma* strains were selected because, despite the *T. asperellum* strain T203 and the T-78 of *T. harzianum* have been demonstrated to induce resistance to RKN in susceptible tomato (Sharon et al., 2009; Martínez-Medina et al., 2017), they are not currently approved in Europe. The strains used in this study are approved in Europe for the management of some fungal plant pathogens, and there are commercial formulates based on these strains available for producers in Spain. The viability of the inoculum was assessed by serial dilution from the commercial formulate and plating onto PDA, and the number of colony forming units were counted after 24 h of incubation at 25°C in the dark.

Second stage juveniles (J2) of the avirulent *M. incognita* population Agròpolis and the virulent population Agrovir were used in this study. The Agrovir population was selected from the Agròpolis population after cultivation of tomato grafted onto the resistant tomato rootstock cv. Aligator (Expósito et al., 2019). J2 were obtained from eggs extracted from resistant (Agrovir population) or susceptible (Agròpolis population) tomato roots by blender maceration in a 5% commercial bleach solution (40 g l^–1^ NaOCl) for 10 min (Hussey and Barker’s, 1973). The suspension was passed through a 74-μm aperture sieve, and the eggs were collected on a 25-μm sieve. Eggs were placed on Baermann trays (Whitehead and Hemming, 1965) and incubated at 25 ± 2°C. J2 were collected daily for 7 days using a 25-μm sieve and stored at 9°C unless used.

### Induction of Systemic Plant Resistance to an Avirulent *M. incognita* Population by *T. asperellum* T34 and *T. harzianum* T22

Tomato and cucumber were grown in a split-root system, following the procedure described in Ghahremani et al. (2019), in which the plant root is divided into two halves transplanted in two adjacent pots: the inducer, inoculated with the antagonist, and the responder, inoculated with the nematode. The main root of 5-day-old seedlings was excised, and plantlets were individually transplanted into seedling trays containing sterile vermiculite and maintained in a growth chamber at 25 ± 2°C with a 16/8 h (light/dark) photoperiod for 2 weeks for cucumber and 3 weeks for tomato plants. Afterward, plantlets were transferred to the split-root system by splitting roots into two halves planted in two adjacent 200-cm^3^ pots filled with sterilized sand. The inducer part of the root was inoculated with a suspension of 10^5^ cfu of *T. harzianum* T22 (T22) or *T. asperellum* T34 (T34) just before transplanting. This fungal dosage was selected because it was the same at which *P. chlamydosporia* induced resistance in tomato (Ghahremani et al., 2019). One week later, the responder part of the root was inoculated with the avirulent *M. incognita* population Agròpolis at a rate of 1 J2 cm^–3^ of soil (RKN). Five treatments were assessed: (i) the inducer inoculated with T22 and the responder with the nematode (T22-RKN), (ii) the inducer inoculated with T34 and the responder with the nematode (T34-RKN), (iii) the inducer non-inoculated with any fungal strain (None) and the responder inoculated with the nematode (None-RKN), (iv) the inducer inoculated with T22 and the responder non-inoculated (T22-None), (v) the inducer inoculated with T34 and the responder non-inoculated (T34-None), and (vi) neither inducer nor responder inoculated (None–None). Treatments (i)–(iii) served to assess the capability of each *Trichoderma* strain to induce plant resistant against the nematode, and treatments (iv)–(vi) were included to assess the effect of each *Trichoderma* strain on plant development. The non-inoculated inducer or responder parts of the root received the same volume of water than those inoculated with the fungal strains or the nematode. Each treatment was replicated 10 times. The plants were maintained in a growth chamber at 25 ± 2°C and photoperiod of 16-/8-h light/dark in a completely randomized design for 40 days. The plants were irrigated as needed and fertilized with Hoagland solution twice per week. Soil temperatures were recorded daily at 30-min intervals with a PT100 probe (Campbell Scientific, Ltd.) placed in the pots at a depth of 4 cm. At the end of the experiments, the foliar surface area of each single plant was measured with a Li-3100 AREA ETER (LI-COR, Inc., Lincoln, NE, United States). Afterward, the aboveground part of each plant was oven dried at 70°C for 2 days, and the dry shoot weight was recorded. The fresh weight of the inducer and responder part of the root system was also recorded. The number of egg masses produced in the responder part of the roots inoculated with the nematode was counted after being stained with a 0.01% erioglaucine solution for 45 min (Omwega et al., 1988). After that, the nematode eggs were extracted from the responder part of the roots by blender maceration in a 10% commercial bleach solution (40 g l^–1^ NaOCl) for 10 min following the Hussey and Barker’s (1973) procedure and counted.

### Combined Effect of *T. asperellum* T34 and Tomato-Resistant Germplasm to an Avirulent *M. incognita* Population

Resistant tomato cv. Monika and susceptible cv. Durinta plants were germinated as previously stated and grown in a growth chamber at 25 ± 2°C and photoperiod of 16/8 h light/dark. Three leaves stage plants were transferred to 200-cm^3^ pots filled with sterilized sand. The experiment was composed by the following treatments: (i) susceptible tomato plants inoculated with 1 J2 cm^–3^ of soil, (ii) susceptible tomato plants inoculated with T34 7 days before nematode inoculation, (iii) resistant tomato plants inoculated with 1 J2 cm^–3^ of soil, and (iv) resistant tomato plants inoculated with T34 7 days before nematode inoculation. The *T. asperellum* T34 was applied at the rate recommended by the manufacturer, 0.01 g l^–1^ of soil as liquid suspension (2 × 10^6^ cfu per plant). Each treatment was replicated 15 times. Plants were maintained in a growth chamber at the same conditions for 40 days. At the end of the experiment, roots were processed as previously described before the number of egg masses and eggs were counted.

### Combined Effect of *T. asperellum* T34 and Tomato-Resistant Germplasm to a Virulent *M. incognita* Population and Effect of T34 on Natural Nematode Antagonism by *P. chlamydosporia*

Plants of the resistant tomato cv. Monika and the susceptible cv. Durinta supplied by Hishtil Gelpi Spain were used for the experiment. The experiment was conducted with soil taken from two sites located at the Tarragona Province (northeastern Spain), M10.23 and M10.55, where vegetables are commercially produced under organic standards in plastic greenhouse. The site M10.23 was a loam soil (45% sand, 40% silt, and 15% clay), pH 8.3, 5.8% organic matter (*w*/*w*), and 276 μS cm^–1^ electric conductivity. The site M10.55 was a sandy clay loam soil (68% sand, 0% silt, and 32% clay), pH 8.1, 2.5 organic matter (*w*/*w*), and 1,069 μS cm^–1^ electric conductivity. Both soils were previously characterized as suppressive to *Meloidogyne*, with *P. chlamydosporia* as the only fungal species recovered from RKN-parasitized eggs (Giné et al., 2016). Each soil was mixed with steam-sterilized sand at a ratio of 1:1 (dry w/dry w), to avoid soil compaction and to improve plant growth, and served as substrate for cropping tomato plants in 3-l pots. The population density of *Meloidogyne* J2 in the soil mixture was determined by counting the nematodes extracted from three 500-cm^3^ samples of each soil mixture by Baermann trays (Whitehead and Hemming, 1965) and incubated at 27 ± 2°C for 1 week. The experiment consisted of four treatments per soil: (i) susceptible tomato plants inoculated in the seedling tray with T34 7 days before transplanting and also just after transplanting and with J2 of the virulent *M. incognita* Agrovir population to achieve 1 J2 cm^–3^ of mixed soil per pot, (ii) susceptible tomato plants inoculated with the virulent nematode population, (iii) resistant tomato plants inoculated in the seedling tray with T34 7 days before transplanting and just after transplanting and with the virulent nematode population, and (iv) resistant tomato plants inoculated with the virulent nematode population. The *T. asperellum* T34 was applied at the dose recommended by the manufacturer as liquid suspension, 0.5 g of T34 m^–2^ of seedling tray before transplanting (1.9 × 10^6^ cfu per plantlet) and 0.01 g l^–1^ of soil (3 × 10^7^ cfu per plant) just after transplanting. Each treatment was replicated 15 times per each soil (M10.23 and M10.55). Plants were maintained in a greenhouse for 40 days. In addition, three plants of each tomato cultivar growing in sterilized sand and inoculated with T34 at the same dose and timing were included to compare the ability of *T. asperellum* to colonize roots in non-sterilized mixed soil versus sterilized sand. At the end of the experiment, three egg masses per plant were taken for quantification of fungal egg parasitism as described in Giné et al. (2016). Afterward, eggs were extracted from roots and counted following the procedure previously described.

The detection and quantification of *T. asperellum* in tomato roots and in M10.23 and M10.55 soils were estimated using the TaqMan-quantitative PCR (qPCR) protocol specifically designed for this fungus by Gerin et al. (2018). Root colonization of plants grown in the M10.23 and M10.55 soils was estimated from three biological replicates per treatment. Each biological replicate consisted of a pool of 3-g, 1-g root per each of three plants. For plants cultivated in sterilized sand, each plant was considered an independent biological replicate. For soil replicates, the pooled soil from three independent pots per treatment was used. DNA extraction from roots was carried out as in Lopez-Llorca et al. (2010), while DNA was extracted from soil samples using the DNeasy PowerLyzer PowerSoil Kit (Qiagen) following the manufacturer’s instructions. All DNA samples were quantified using Qubit dsDNA BR assay kit (Thermo Fisher Scientific). qPCR reactions were performed using the Sso AdvancedTM Universal Probes Supermix (Bio-Rad Laboratories, Hercules, CA, United States) in a final volume of 20 μl containing 40 ng of total DNA, 250 nM of each primer (5′ to 3′ direction) Ta_rpb2_fw (GGAGGTCGTTGAGGAGTACGAA) and Ta_rpb2_rev_3 (TTGCAGATAGGATTTACGACGAGT) and 150 nM of Ta_rpb2_probe (FAM-CGCTGAGGTATCCCCATGCGACA-BHQ1) (Gerin et al., 2018). Negative controls containing sterile water instead of DNA were included. Reactions were performed in duplicate in a 7900HT Fast Real-Time PCR System thermocycler (Applied Biosystems) using the following thermal cycling conditions: initial denaturation step at 95°C for 2 min, then 40 cycles at 95°C for 5 s, and 64.5°C for 30 s. Genomic DNA dilutions of the *T. asperellum* T34 were used to define a calibration curve from 10 pg to 100 ng. The specificity of the PCR amplicons was verified by agarose gel electrophoresis. *T. asperellum* DNA biomass was referred to the total DNA biomass (40 ng).

### Statistical Analysis

Statistical analyses were performed using the JMP software v8 (SAS Institute, Inc., Cary, NC, United States). Both data normality and homogeneity of variances were assessed. When confirmed, a paired comparison using the Student’s *t*-test was done, or Dunnett’s test for multiple comparisons with a control. Otherwise, paired comparison was done using the non-parametric Wilcoxon test or multiple comparison using the Kruskal–Wallis test and groups separated by Dunn’s test (*P* ≤ 0.05).

## Results

### Induction of Systemic Plant Resistance to an Avirulent *M. incognita* Population by *T. asperellum* T34 and *T. harzianum* T22

**FIGURE 1:**
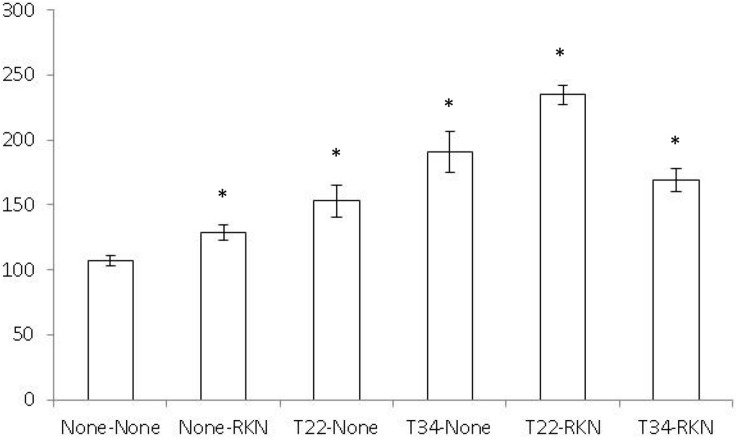
Cucumber leaf area surface (cm^2^) in a split-root system experiment conducted in two adjacent 200-ml pots (inducer–responder) in which the inducer part of the root was non-inoculated (None-) or inoculated with 10^5^ cfu of *Trichoderma harzianum* T22 (T22-) or *T. asperellum* T34 (T34-) just before transplanting and the responder part of the root was non-inoculated (-None) or inoculated at a rate of 1 J2 cm^–3^ of soil of the avirulent *Meloidogyne incognita* population Agròpolis (-RKN) 1 week after fungal inoculation. Each value is mean (column) of 10 replications and the standard error (bar). Column with asterisk differ (*P* < 0.05) from the treatment None–None according to the Dunnett’s test.

The split-root system did not influence tomato or cucumber root development since root fresh weight did not differ between the two halves of the split-root system of the None–None treatment (*P* < 0.05) (data not shown). Both tomato shoot dry biomass and leaf surface area did not differ (*P* < 0.05) between treatments (data not shown). In cucumber, shoot dry biomass did not differ, but the leaf surface area of the None–None treatment was lower (*P* < 0.05) than the remaining ones (Figure 1).

**FIGURE 2:**
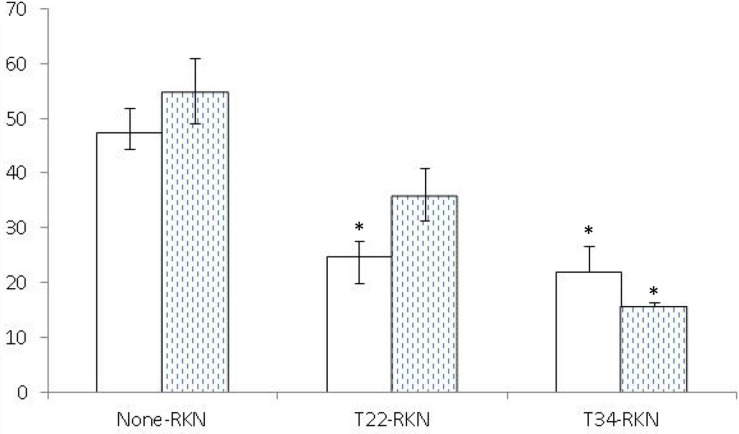
Number of eggs (× 10^3^) (white column) and egg masses (spotted column) produced by the avirulent *M. incognita* population Agròpolis (-RKN) in the responder part of the root of the susceptible tomato cv. Durinta in a split-root system experiment conducted in two adjacent 200-ml pots (inducer–responder) in which the inducer part of the root was non-inoculated (None-) or inoculated with 10^5^ cfu of *T. harzianum* T22 (T22-) or *T. asperellum* T34 (T34-) just before transplanting and the responder part of the root was non-inoculated (-None) or inoculated at a rate of 1 J2 cm^–3^ of soil of the avirulent *M. incognita* population Agròpolis 1 week after fungal inoculation. Each value is mean of 10 replications and the standard error (bar). Column for each variable with asterisk differ (*P* < 0.05) from the treatment None-RKN according to the Dunnett’s test.

**FIGURE 3:**
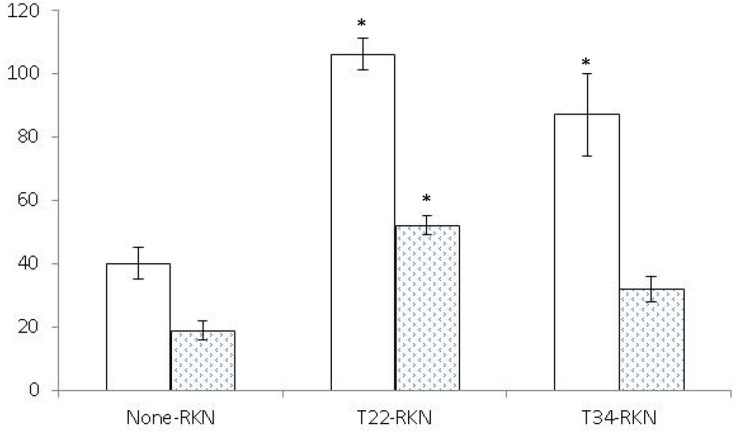
Number of eggs (× 10^2^) (white column) and egg masses (spotted column) produced by the avirulent *M. incognita* population Agròpolis (-RKN) in the responder part of the root of the susceptible cucumber cv. Dasher II in a split-root system experiment conducted in two adjacent 200-ml pots (inducer–responder) in which the inducer part of the root was non-inoculated (None-) or inoculated with 10^5^ cfu of *T. harzianum* T22 (T22-) or *T. asperellum* T34 (T34-) just before transplanting and the responder part of the root was non-inoculated (-None) or inoculated at a rate of 1 J2 cm^–3^ of soil of the avirulent *M. incognita* population Agròpolis 1 week after fungal inoculation. Each value is mean of 10 replications and the standard error (bar). Column for each variable with asterisk differ (*P* < 0.05) from the treatment None-RKN according to the Dunnett’s test.

Both *Trichoderma* strains induced systemic resistance in tomato (Figure 2) but not in cucumber (Figure 3). *Trichoderma asperellum* T34 reduced both nematode infectivity and reproduction (*P* < 0.05) by 71 and 54%, respectively. Meanwhile, *T. harzianum* T22 suppressed nematode reproduction by 48%, but did not affect nematode infectivity (*P* < 0.05). For cucumber, the number of egg masses in the responder part of the root of the T22-RKN treatment was 2.7 times higher (*P* < 0.05) than in the None-RKN treatment, and the number of eggs in the responder part of the root of T22-RKN and T34-RKN treatments was 2.7 and 2.2 times higher (*P* < 0.05) than in the None-RKN treatment, respectively.

### Combined Effect of *T. asperellum* T34 and Tomato-Resistant Germplasm to an Avirulent *M. incognita* Population

The infectivity and reproduction of the avirulent *M. incognita* population Agròpolis in the non-inoculated T34 resistant tomato were 97.7 and 97.2% lower (*P* < 0.05) than in the susceptible cultivar. For resistant tomato inoculated with T34, we observed a reduction of 98.2 and 98.7%, respectively, compared to the susceptible cultivar treated with T34 (*P* < 0.05).

**FIGURE 4:**
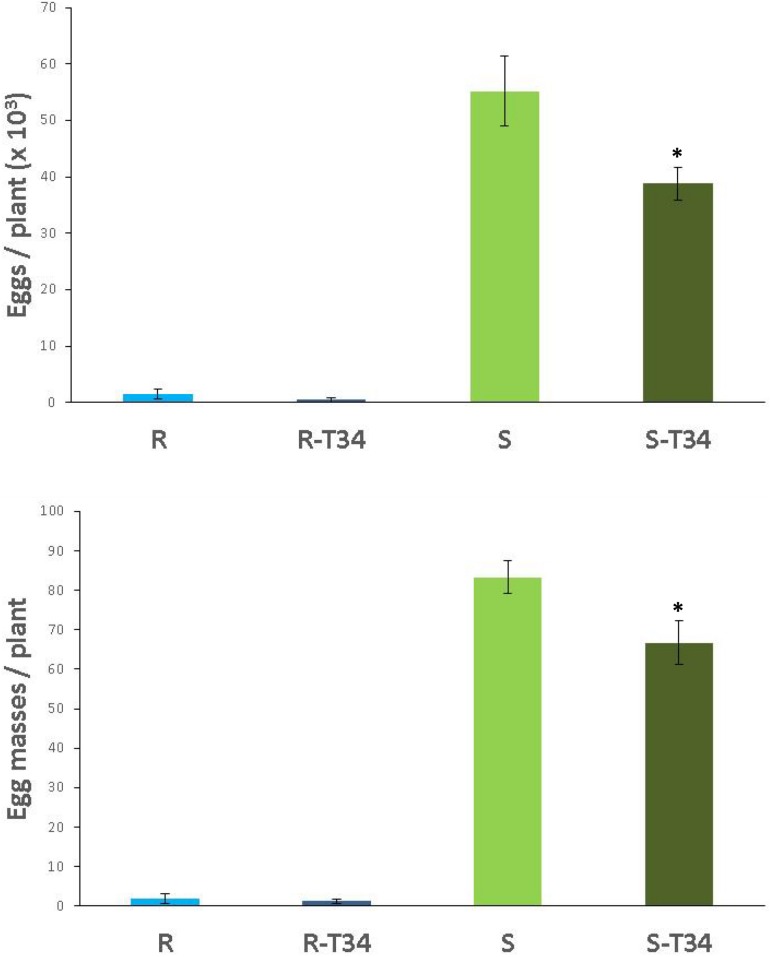
Number of eggs (× 10^3^) and egg masses produced by the avirulent *M. incognita* population Agròpolis in the resistant tomato cv. Monika (R) and the susceptible cv. Durinta (S) cultivated in 200-ml pots inoculated with *Trichoderma asperellum* T34 (T34) at a rate of 0.01 g l^–1^ of soil (2 × 10^6^ cfu per plant) just after transplanting and 7 days before inoculation with 1 J2 cm^–3^ of soil. Each value is mean (column) of 15 replications and the standard error (bar). Column with asterisk indicate differences (*P* < 0.05) between treatments within each tomato cultivar according to the Wilcoxon test.

The number of egg masses and eggs of *M. incognita* produced in the susceptible tomato plants inoculated with T34 were 20 and 30% lower (*P* < 0.05) than those observed in the non-inoculated susceptible plants. Regarding the resistant tomato, non-statistical differences (*P* < 0.05) were found between T34-inoculated and non-inoculated plants. Nonetheless, fewer egg masses and eggs per plant were recorded in the resistant tomato inoculated with T34 than in the non-inoculated (Figure 4).

### Combined Effect of *T. asperellum* T34 and Resistant Tomato to a Virulent *M. incognita* Population and Effect of T34 on Natural Nematode Antagonism by *P. chlamydosporia*

**FIGURE 5:**
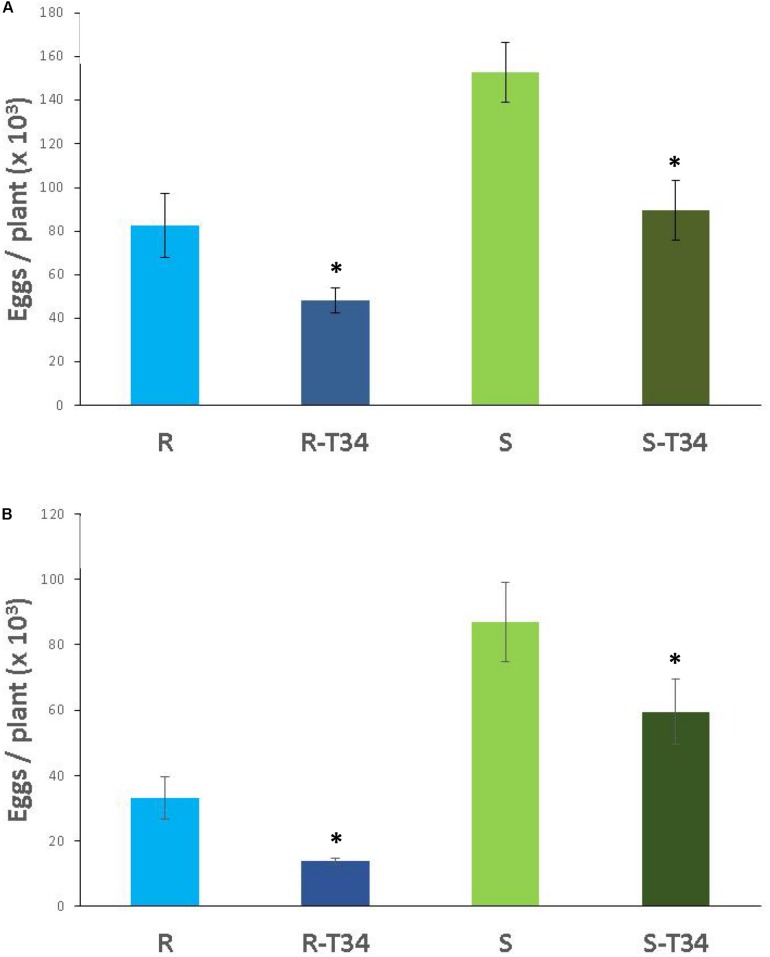
Number of eggs (× 10^3^) produced by the virulent *M. incognita* population Agrovir in the resistant tomato cv. Monika (R) and the susceptible cv. Durinta (S) inoculated with *Trichoderma asperellum* T34 (T34) in the seedling trays at a rate of 0.5 g m^–2^ (1.9 × 10^6^ cfu per plantlet) 7 days before transplanting, and at rate of 0.01 g l^–1^ of soil M10.23 **(A)** or M10.55 **(B)** at transplanting in 3-L pots (3 × 10^7^ cfu per plant) and inoculated with J2 to achieve a rate of 1 J2 cm^–3^ of soil. Each value is mean (column) of 15 replications and the standard error (bar). Column with asterisk indicate differences (*P* < 0.05) between treatments within each tomato cultivar according to the Wilcoxon test.

In soil M10.23, resistant tomato plants presented a 46% reduction of eggs compared to susceptible tomato plants (*P* < 0.05) independently of T34 treatment. In both tomato cultivars, the nematode produced 41% fewer eggs in T34-inoculated than in non-inoculated plants (Figure 5A). *P. chlamydosporia* was the only fungal species isolated from parasitized eggs. The percentage of parasitized eggs ranged from 21 to 28% and did not differ (*P* < 0.05) between tomato cultivars or between T34-inoculated and non-inoculated plants.

In soil M10.55, the nematode produced 77% fewer eggs (*P* < 0.05) in the resistant than in the susceptible tomato cultivar inoculated with T34, and 62% fewer eggs (*P* < 0.05) in the non-T34 inoculated resistant than susceptible tomato plants. The nematode produced 58% fewer (*P* < 0.05) eggs in T34-inoculated than in non-inoculated resistant tomato, and 31.6% fewer eggs in susceptible T34-inoculated tomato than in non-inoculated (Figure 5B). The percentage of parasitized eggs ranged from 25.4 to 28.9% and did not differ between tomato cultivars or T34-inoculated and non-inoculated plants. *P. chlamydosporia* was isolated from 90% of the total parasitized eggs, *T. asperellum* from 5%, and the remaining 5% were parasitized by an unidentified fungal species.

The standard curve for qPCR obtained by representing the cycle threshold (Ct) against log of 10-fold serial dilution of DNA from *T. asperellum* T34 was accurate and reproducible to estimate the DNA concentration of this fungal species (*y* = −3.1479*x* + 24.79; *R*^2^ = 0.9956). The DNA concentration in the M10.23 and M10.55 soil samples was very low since Ct values were higher than 35 cycles. Regarding the root samples, the DNA concentration was also very low except for two biological replicates of tomato cultivated in soil M10.55, one biological replicate of cv. Durinta inoculated with T34 and one of cv. Monika non-inoculated with T34 (Supplementary Table S1). Regarding the tomato plants cultivated in sterilized sand inoculated with T34, the majority of the Ct values was below 35 cycles, and the *T. asperellum* DNA concentration was estimated from those replicates. After analyzing 40 ng of DNA extracted from tomato roots, the estimated DNA content for *T. asperellum* did not differ (*P* < 0.05). It was 0.067 ± 0.018 ng in cv. Durinta and 0.087 ± 0.027 ng in cv. Monika.

## Discussion

This study provides evidence for the ability of two commercial *Trichoderma-*based formulates, T34 Biocontrol (*T. asperellum* strain T34) and Trianum P (*T. harzianum* strain T22), to induce systemic resistance in tomato against *M. incognita.* Systemic induction of plant defense mechanisms in tomato to RKN by different species and/or strains of *Trichoderma*, i.e., *T. asperellum* strain 203, *T. atroviride* strain T11, and *T. harzianum* strain T78, has been proven (Sharon et al., 2009; de Medeiros et al., 2017; Martínez-Medina et al., 2017). Martínez-Medina et al. (2017) studied the hormonal regulation pathways and proposed a three-phase model, that is, an early induction of the salicylic acid pathway suppressing RKN infection, a second phase mediated by jasmonic acid induction suppressing RKN reproduction and fecundity, and a final salicylic acid induction that affects root infection by the next J2 generation. Interestingly, the results of this study show that the resistance is also induced in tomato bearing the *Mi-1.2* resistance gene. The effect of combining *R*-genes with induced resistance by *Trichoderma* was more evident against the virulent nematode population than the avirulent one, which was highly suppressed by the *Mi-1.2* resistance gene although relatively less infection (34%) and reproduction (67%) was recorded in relation to the non-inoculated plants. Primed plants by *Trichoderma* can suppress the virulent nematode reproduction about 50%. Then, the resistance induced by *Trichoderma* is additive to that provided by the *Mi-1.2* gene. Thus, primed plants could be used as an additional sustainable tool to manage virulent nematode populations and also be useful for suppressing RKN species non-affected by the *M1.2* resistance gene such as *Meloidogyne hapla* (Liu and Williamson, 2006) and *Meloidogyne enterolobii* (Brito et al., 2007). Nonetheless, some -omic studies should be conducted to know the gene regulation and the physiological changes induced by the fungus in resistant tomato plants to foresee the possible medium to long-term consequences of using both types of resistance. If the additive effect is due to an overexpression of defense mechanisms usually expressed in *R*-gene-resistant plants, the probability for selecting virulent nematodes could be higher than if additional defense mechanisms are activated.

Interestingly, we found that the induction of resistance to *M. incognita* by both *Trichoderma* strains vary between plant species. Some reports have demonstrated the capability of *Trichoderma* strains to induce resistance in cucumber against several microbial plant pathogens (Khan et al., 2004; Shoresh et al., 2005; Segarra et al., 2007; Alizadeh et al., 2013; Sabbagh et al., 2017; Yuan et al., 2019). Segarra et al. (2007) found that *T. asperellum* T34 increased the concentration of salicylic acid and jasmonic acid in cotyledons of cucumber between 3 and 48 h suppressing *Pseudomonas syringae* pv. *lachrymans* inoculated 24 h after fungal inoculation. That experiment used a different inoculation regime, including a higher concentration of fungal spores, and thus, it is possible that the dosage used in the present work is sufficient to induce resistance in tomato but not in cucumber. *P. chlamydosporia* also induced systemic plant resistance against *M. incognita* in tomato but not in cucumber inoculated with 10^5^ viable chlamydospores (Ghahremani et al., 2019). Increasing the inoculum density of *Trichoderma* per cucumber plant could modify this result and should be investigated. In addition to that, other causes could be responsible for the lack of induction of plant resistance to RKN. Recently, Chen et al. (2018) have reported that vanillic acid, a root exudate of cucumber, influences the fungal community in the rhizosphere and the abundance of *Trichoderma* and *Fusarium* species depending on the vanillic acid concentration. In our study, both *Trichoderma* strains increased the nematode reproduction in cucumber compared to the non-fungal-inoculated plants. As such, we cannot discard the possibility that *Trichoderma* might increase nematode susceptibility on cucumber plants. Further studies are needed to elucidate this.

T34 did not affect *P. chlamydosporia* egg parasitism that naturally occurred in soils M10.23 and M10.55. The ability of some *Trichoderma* spp. to parasitize nematode eggs and juveniles has been proven, and the mechanisms involved have been studied (summarized in Sharon et al., 2011; Szabó et al., 2012). The *T. asperellum* T34 used in this study can parasitize individual eggs in *in vitro* conditions (data not shown), but it was only isolated from 5% of the total *M. incognita* parasitized eggs produced in tomato cultivated in soil M10.55 inoculated with T34. This reduced ability of *Trichoderma* for parasitizing RKN eggs in comparison to other fungal egg parasites naturally occurring in soil can explain the lack of references regarding isolation of *Trichoderma* spp. from RKN eggs in vegetable growing areas from Spain (Olivares and López-Llorca, 2002; Verdejo-Lucas et al., 2002, 2013; Giné et al., 2012).

It is known that *Trichoderma* spp. strains can act as nematode antagonists affecting J2 motility, nematode development, egg hatching, nematode reproduction, and disease severity (summarized in Sharon et al., 2009; Wann et al., 2016), and also inducing resistance against RKN in susceptible tomato cultivars (Sharon et al., 2009; de Medeiros et al., 2017; Martínez-Medina et al., 2017). This study provides new evidence of the ability of some additional *Trichoderma* strains to induce resistance to RKN in susceptible tomato and demonstrate for the first time the ability to induce resistance in tomato carrying the *Mi-1.2* gene and that this resistance is additive to that provided by the *R-*gene against a virulent nematode population.

## Conclusion

This study proves that the strains T34 of *T. asperellum* and T22 of *T. harzianum* induce resistance against *M. incognita* in tomato but not in cucumber, at least under our experimental conditions. Resistance conferred by the *Mi-1.2* resistance gene and that induced by T34 in tomato is additive. Finally, T34 does not affect the egg parasitism by the naturally occurring *P. chlamydosporia*. To foresee the potential selection for nematode virulence, future studies are needed to understand the genes related and the physiological changes involved in inducing resistance in tomato plants bearing the *Mi-1.2* gene. Moreover, the compatibility of commercial *Trichoderma* formulates with nematode antagonists that occurs naturally should be studied in deep to avoid potential detrimental effects.

## Data Availability Statement

All datasets generated for this study are included in the article/Supplementary Material.

## Author Contributions

FS and NE conceived, designed, and supervised the experiments, the data collection, and analyses. MP performed the split-root experiments and analyzed the data. AF, MF, and PV performed the experiments combining plant germplasm with T34 in different soils and analyzed the data. NE, ES, MF, and AF performed the molecular analysis. TG provided reagents, materials, and advice. NE, ES, TG, and FS wrote the manuscript.

## Conflict of Interest

The authors declare that the research was conducted in the absence of any commercial or financial relationships that could be construed as a potential conflict of interest.

## Supplementary Material

TABLE S1 Threshold cycle (Ct) values, were obtained for quantifying *T. asperellum* using the primers and probe designed by Gerin et al. (2018) for every sample and technical replicates. The detection was carried out from roots of the susceptible tomato cv. Durinta, and the resistant cv. Monika non-inoculated or inoculated with *T. asperellum* T34 (T34) cultivated in two suppressive soils (M10.23 and M10.55) or in sterilized sand. The detection of the fungus was also assessed in soils M10.23 and M10.55. Root colonization of plants grown in the M10.23 and M10.55 soils was estimated from three biological replicates per treatment. Each biological replicate consisted of a pool of 3-g, 1-g root per each of three plants. For plants cultivated in sterilized sand, each plant was considered an independent biological replicate. For soil replicates, the pooled soil from three independent pots per treatment was used.

## References

[B1] Alizadeh H. Behboudi K. Ahmadzadeh M. Javan-Nikkhah M. Zamioudis C. Pieterse C. M. J. (2013). Induced systemic resistance in cucumber and *Arabidopsis thaliana* by the combination of *Trichoderma harzianum* Tr6 and *Pseudomonas* sp. Ps14. *Biol. Control.* 65 14–23. 10.1016/j.biocontrol.2013.01.009

[B2] Brito J. A. Stanley J. D. Kaur R. Cetintas R. di Vito M. Thies J. A. (2007). Effects of the Mi-1, N and *Tabasco* genes on infection and reproduction of *Meloidogyne mayaguensis* on tomato and pepper genotypes. *J. Nematol.* 39 327–332. 19259507 PMC2586510

[B3] Chen S. Yu H. Zhou X. Wu F. (2018). Cucumber (*Cucumis sativus* L.) seedling *Rhizosphere trichoderma* and *Fusarium* spp. Communities altered by Vanillic acid. *Front. Microbiol.* 9:2195. 10.3389/fmicb.2018.02195 30283420 PMC6157394

[B4] Cortada L. Sorribas F. J. Ornat C. Kaloshian I. Verdejo-Lucas S. (2008). Variability in infection and reproduction of *Meloidogyne javanica* on tomato rootstocks with the Mi resistance gene. *Plant Pathol.* 57 1125–1135. 10.1111/j.1365-3059.2008.01906.x

[B5] de Medeiros H. A. de Araújo Filho J. V. de Freitas L. G. Castillo P. Rubio M. B. Hermosa R. (2017). Tomato progeny inherit resistance to the nematode *Meloidogyne javanica* linked to plant growth induced by the biocontrol fungus *Trichoderma atroviride*. *Sci. Reps.* 7:40216. 10.1038/srep40216 28071749 PMC5223212

[B6] Djian-Caporalino C. (2012). Root-knot nematode (*Meloidogyne* spp.), a growing problem in French vegetable crops. *EPPO Bull.* 42 127–137. 10.1111/j.1365-2338.2012.02530.x

[B7] Expósito A. García S. Giné A. Escudero N. Sorribas F. J. (2019). Cucumis metuliferus reduces *Meloidogyne incognita* virulence against the Mi1.2 resistance gene in a tomato–melon rotation sequence. *Pest Manag. Sci.* 77 1902–1910. 10.1002/ps.5297 30536835

[B8] Ferreira P. A. Ferraz S. Lopes E. A. de Freitas L. G. (2008). Parasitismo de ovos de *Meloidogyne exigua* por fungos nematófagos e estudo da compatibilidade entre os isolados fúngicos. *Revista Trópica* 3 15–21.

[B9] Gerin D. Pollastro S. Raguseo C. De Miccolis Angelini R. M. Faretra F. (2018). A ready-to-use single- and duplex-TaqMan-qPCR assay to detect and quantify the biocontrol Agents *Trichoderma asperellum* and *Trichoderma gamsii*. *Front. Microbiol.* 9:2073. 10.3389/fmicb.2018.02073 30233545 PMC6127317

[B10] Ghahremani Z. Escudero N. Saus E. Gabaldón T. Sorribas F. J. (2019). Pochonia chlamydosporia Induces plant-dependent systemic resistance to *Meloidogyne incognita*. *Front. Plant Sci.* 10:945. 10.3389/fpls.2019.00945 31456811 PMC6700505

[B11] Giné A. Bonmatí M. Sarro A. Stchiegel A. Valero J. Ornat C. (2012). Natural occurrence of fungal egg parasites of root-knot nematodes, *Meloidogyne* spp. in organic and integrated vegetable production systems in Spain. *Biocontrol* 58 407–416. 10.1007/s10526-012-9495-6

[B12] Giné A. Carrasquilla M. Martínez-Alonso M. Gaju N. Sorribas F. J. (2016). Characterization of soil suppressiveness to root-knot nematodes in organic horticulture in plastic greenhouse. *Front. Plant Sci.* 7:164. 10.3389/fpls.2016.00164 26925080 PMC4756147

[B13] Giné A. González C. Serrano L. Sorribas F. J. (2017). Population dynamics of *Meloidogyne incognita* on cucumber grafted onto the cucurbita hybrid RS841 or ungrafted and yield losses under protected cultivation. *Eur. J Plant. Pathol.* 148 795–805. 10.1007/s10658-016-1135-z

[B14] Giné A. López-Gómez M. Vela M. D. Ornat C. Talavera M. Verdejo-Lucas S. (2014). Thermal requirements and population dynamics of root-knot nematodes on cucumber and yield losses under protected cultivation. *Plant Pathol.* 6 1446–1453. 10.1111/ppa.12217

[B15] Giné A. Sorribas F. J. (2017). Quantitative approach for the early detection of selection for virulence of *Meloidogyne incognita* on resistant tomato in plastic greenhouses. *Plant Pathol.* 66 1338–1344. 10.1111/ppa.12679

[B16] Hallman J. Meressa B. H. (2018). “Nematode parasites of vegetables,” in *Plant Parasitic Nematodes in Subtropical and Tropical Agriculture*, eds Sikora R. A. Coyne D. Hallman J. Timper P. (Wallingford: CABI International), 346–410. 10.1079/9781786391247.0346

[B17] Hallmann J. Davies K. G. Sikora R. (2009). “Biological control using microbial pathogens, endophytes and antagonists,” in *Root-knot Nematodes*, eds Perry R. N. Moens M. Starr J. L. (Wallingford: CABI international), 380–411. 10.1079/9781845934927.0380

[B18] Hussey R. S. Barker K. R. (1973). A comparison of methods of collecting inoculate of *Meloidogyne* spp. including a new technique. *Plant Dis. Rep.* 57 1025–1028.

[B19] Jones J. T. Haegeman A. Danchin E. G. Gaur H. S. Helder J. Jones M. G. (2013). Top 10 plant-parasitic nematodes in molecular plant pathology. *Mol. Plant Pathol.* 14 946–961. 10.1111/mpp.12057 23809086 PMC6638764

[B20] Khan J. Ooka J. J. Miller S. A. Madden L. V. Hoitink H. A. J. (2004). Systemic resistance induced by *Trichoderma hamatum* 382 in cucumber against Phytophthora crown rot and leaf blight. *Plant Dis.* 88 280–286. 10.1094/PDIS.2004.88.3.280 30812360

[B21] Kok C. J. Papert A. Hok-A-Hin C. H. (2001). Microflora of *Meloidogyne* egg masses: species composition, population density and effect on the biocontrol agent *Verticillium chlamydosporium* (Goddard). *Nematology* 3 729–734. 10.1163/156854101753625236

[B22] Liu Q. L. Williamson V. M. (2006). Host-specific pathogenicity and genome differences between inbred strains of *Meloidogyne hapla*. *J. Nematol.* 38 158–164. 19259441 PMC2586434

[B23] López-Gómez M. Giné A. Vela M. D. Ornat C. Sorribas F. J. Talavera M. (2014). Damage function and thermal requirements of *Meloidogyne javanica* and *Meloidogyne incognita* on watermelon. *Ann. Appl. Biol.* 165 466–473.

[B24] Lopez-Llorca L. V. Gómez-Vidal S. Monfort E. Larriba E. Casado-Vela J. Elortza F. (2010). Expression of serine proteases in egg-parasitic nematophagous fungi during barley root colonization. *Fungal Genet. Biol.* 47 342–351. 10.1016/j.fgb.2010.01.004 20097301

[B25] Martínez-Medina A. Fernandez I. Lok G. B. Pozo M. J. Pieterse C. M. Van Wees S. C. (2017). Shifting from priming of salicylic acid- to jasmonic acid-regulated defences by *Trichoderma* protects tomato against the root knot nematode *Meloidogyne incognita*. *New Phytol.* 213 1363–1377. 10.1111/nph.14251 27801946

[B26] Nyczepir A. P. Thomas S. H. (2009). “Current and future management strategies in intensive crop production systems,” in *Root-knot Nematodes*, eds Perry R. N. Moens M. Starr J. L. (Wallingford: CABI international), 412–443. 10.1079/9781845934927.0412

[B27] Olivares C. M. López-Llorca L. V. (2002). Fungal egg-parasites of plant-parasitic nematodes from Spanish soils. *Rev. Iberoam. Micol.* 19 104–110. 12828513

[B28] Omwega C. Thomason I. J. Roberts P. A. (1988). A non-destructive technique for screening bean germ plasm for resistance to *Meloidogyne incognita*. *Plant Dis.* 72 970–972.

[B29] Puertas A. I. de la Noval B. M. Martínez B. Miranda I. Fernández F. E. Hidalgo L. (2006). Interacción de *Pochonia chlamydosporia* var. catenulata con *Rhizobium* sp., *Trichoderma harzianum* y Glomus clarum en el control de *Meloidogyne incognita*. *Revista de Protección Vegetal* 21 80–89.

[B30] Roberts P. A. (2002). “Concepts and consequences of resistance,” in *Plant Resistance to Parasitic Nematodes*, eds Starr J. L. Cook R. Bridge J. (Wallingford: CABI International), 23–41. 10.1079/9780851994666.0023

[B31] Ros-Ibáñez C. Robertson L. Martínez-Lluch M. Cano-García A. Lacasa-Plasencia A. (2014). Development of virulence to *Meloidogyne incognita* on resistant pepper rootstocks. *Span. J. Agric. Res.* 12 225–232.

[B32] Sabbagh S. K. Roudini M. Panjehkeh N. (2017). Systemic resistance induced by *Trichoderma harzianum* and *Glomus mossea* on cucumber damping-off disease caused by *Phytophthora melonis*. *Arch. Phytopathol. Pfl* 50 375–388. 10.1080/03235408.2017.1317953

[B33] Schouten A. (2016). Mechanisms involved in nematode control by endophytic fungi. *Annu. Rev. Phytopathol.* 54 121–142. 10.1146/annurev-phyto-080615-100114 27296146

[B34] Segarra G. Casanova E. Bellido D. Odena M. A. Oliveira E. Trillas I. (2007). Proteome, salicylic acid, and jasmonic acid changes in cucumber plants inoculated with *Trichoderma asperellum* strain T34. *Proteomics* 7 3943–3952. 10.1002/pmic.200700173 17902191

[B35] Seid A. Fininsa C. Mekete T. Decraemer W. Wesemael W. M. L. (2015). Tomato (*Solanum lycopersicum*) and root-knot nematodes (*Meloidogyne* spp.) –a century-old battle. *Nematology* 17 995–1009. 10.1163/15685411-00002935

[B36] Sharon E. Chet I. Bar-Eyal M. Spiegel Y. (2009). “Biocontrol of root-knot nematodes by *Trichoderma –* modes of action,” in *Proceedings of IOBC Meeting on Multitrophic Interactions in Soil*, Vol. 42 (Dijon: IOBC/WPRS Bull), 159–163.

[B37] Sharon E. Chet I. Spiegel Y. (2011). “*Trichoderma* as a biological control agent,” in *Biological Control of Plant-Parasitic Nematodes: Building Coherence Between Microbial Ecology and Molecular Mechanisms, Progress in Biological Control*, eds Davies K. Spiegel Y. (Netherlands: Springer), 183–201. 10.1007/978-1-4020-9648-8_8

[B38] Shoresh M. Yedidia I. Chet I. (2005). Involvement of jasmonic acid/ethylene signaling pathway in the systemic resistance induced in cucumber by *Trichoderma asperellum* T203. *Phytopathology* 95 76–84. 10.1094/PHYTO-95-0076 18943839

[B39] Sorribas F. J. Ornat C. Verdejo-Lucas S. Galeano M. Valero J. (2005). Effectiveness and profitability of the Mi-resistant tomatoes to control root-knot nematodes. *Eur. J. Plant. Pathol.* 111 29–38. 10.1007/s10658-004-1982-x

[B40] Szabó M. Csepregi K. Gálber M. Virányi F. Fekete C. (2012). Control plant-parasitic nematodes with *Trichoderma* species and nematode-trapping fungi: the role of chi18-5 and chi18-12 genes in nematode egg-parasitism. *Biol. Control* 63 121–128. 10.1016/j.biocontrol.2012.06.013

[B41] Talavera M. Sayadi S. Chirosa-Ríos M. Salmerón T. Flor-Peregrín E. Verdejo-Lucas S. (2012). Perception of the impact of root-knot nematode-induced diseases in horticultural protected crops of south-eastern Spain. *Nematology* 14 517–527. 10.1163/156854112x635850

[B42] Thies J. A. (2011). Virulence of *Meloidogyne incognita* to expression of N gene in pepper. *J. Nematol.* 43 90–94. 22791917 PMC3380459

[B43] Verdejo-Lucas S. Blanco M. Talavera M. Stchigel A. M. Sorribas F. J. (2013). Fungi recovered from root-knot nematodes infecting vegetables under protected cultivation. *Biocontrol Sci. Tech.* 23 277–287. 10.1080/09583157.2012.756459

[B44] Verdejo-Lucas S. Cortada L. Sorribas F. J. Ornat C. (2009). Selection of virulent populations of *Meloidogyne javanica* by repeated cultivation of Mi-resistance gene tomato rootstocks under field conditions. *Plant Pathol.* 58 990–998. 10.1111/j.1365-3059.2009.02089.x

[B45] Verdejo-Lucas S. Ornat C. Sorribas F. J. Stchiegel A. (2002). Species of root-knot nematodes and fungal egg parasites recovered from vegetables in Almería and Barcelona. *Spain. J. Nematol.* 34 405–408. 19265964 PMC2620581

[B46] Wann S. B. Borah B. Ahmed R. Gogoi B. Phukon P. Baruah J. (2016). “Isolation, characterization of nematode-controlling bacteria and fungi from nature,” in *Microbial Inoculants in Sustainable Agricultural Productivity, vol. 1: Research Perspectives*, eds Singh D. P. Singh H. B. Prabha R. (New Delhi: Springer India), 271–296.

[B47] Whitehead A. G. Hemming J. R. (1965). A comparison of some quantitative methods of extracting small vermiform nematodes from soil. *Ann. Appl. Biol.* 55 25–38. 10.1111/j.1744-7348.1965.tb07864.x

[B48] Williamson W. M. Roberts P. A. (2009). “Mechanisms and genetics of resistance,” in *Root-knot Nematodes*, eds Perry R. N. Moens M. Starr J. L. (Wallingford: CABI international), 301–325.

[B49] Yuan M. Huang Y. Ge W. Jia Z. Song S. Zhang L. (2019). Involvement of jasmonic acid, ethylene and salicylic acid signaling pathways behind the systemic resistance induced by *Trichoderma longibrachiatum* H9 in cucumber. *BMC Genom.* 20:144. 10.1186/s12864-019-5513-8 30777003 PMC6379975

